# Individual Bilateral Difference of Femur, Tibia, and Leg Rotation: A Clinical Study of 141 Healthy Japanese Individuals Using Computed Tomography

**DOI:** 10.7759/cureus.60750

**Published:** 2024-05-21

**Authors:** Yo Kinami, Norio Yamamoto, Masahiro Horita, Kazuo Fujiwara

**Affiliations:** 1 Department of Orthopedic Surgery, Okayama City Hospital, Okayama, JPN; 2 Department of Epidemiology, Okayama University Graduate School of Medicine, Dentistry and Pharmaceutical Sciences, Okayama, JPN

**Keywords:** japanese, tibia rotation, femur version, malrotation, individual bilateral difference

## Abstract

Background

The malrotation of a femur and tibial fracture after surgery has been described in many articles. However, these studies have not considered individual bilateral differences (IBDs). The IBD of femur and tibial rotation has been identified via computed tomography (CT) in recent American studies. The IBD in rotation should be considered during femur and tibial surgery. However, IBDs in femur and tibial rotation remain unknown in the Japanese population. This study aimed to evaluate the rotation of the femur, knee, tibia, and leg, sex differences, and IBD in rotation among Japanese individuals with healthy bones by using CT analysis.

Materials and methods

In total,141 patients who underwent CT angiography or venography were included (70 men, 71 women; mean age, 44.7 years). The bilateral axial femur, knee, tibia, and leg rotation alignment were independently measured. The distribution, sex, and IBD were analyzed. The IBD in rotation had two statistical factors: absolute bilateral difference (ABD) and relative bilateral difference (RBD).

Results

The mean ABD of femur rotation was 6.5°, and the distribution of ABD of femur rotation ≤15° was 95%. The mean ABD of tibia rotation was 5.1°, and the distribution of ABD of tibia rotation ≤10° was 89%. The RBD of femur rotation was not significantly different between the right and left sides. The RBD of tibia rotation showed a higher mean external rotation of 3.3° on the right side (<0.001). The Pearson correlation coefficients of the femur, knee, tibia, and leg rotation between the right and left sides were high (r= 0.702-0.81; all, p<0.001). All elements of rotation showed significant differences between men and women, whereas the ABD and RBD of all elements showed no significant difference.

Conclusion

The distributions of ABD in femur and tibia rotation supported the previous definition of an acceptable rotation difference between the normal and fractured femur and tibia of ≤15°and ≤10°, respectively. The possibility of higher external rotation on the right side needs to be taken into account during tibial surgery.

## Introduction

The rotation of a fractured femur and tibia is reduced during surgery to a rotation similar to that of the normal femur and tibia. An acceptable rotation of a fractured femur and tibia is defined as ≤15° and ≤10°, respectively, compared with the normal side, to prevent functional impairment [1-10]. In contrast, malrotation of the femur and tibia after surgery has been described in many studies [1-10] using computed tomography (CT) analysis. The malrotation rate is 8.3%-41.7% in femoral shaft fractures [1-5] and 19%-41% in tibial shaft fractures [6-10]. Rotational differences >15° in the femur and >10° in the tibia, compared with the normal side, are considered true deformities, and derotation osteotomy may be indicated [11,12].

These previous studies did not refer to individual bilateral differences (IBDs), although IBDs in femur and tibia rotation have been identified via CT analysis in recent literature [13-16]. Individual bilateral differences have two statistical factors: absolute bilateral difference (ABD) and relative bilateral difference (RBD). The outcomes of one study [13] showed that the mean ABDs of the femur, tibia, and leg rotation are 6.0°, 5.7°, and 9.5°, respectively, and the RBD of the femur, tibia, and whole-leg rotation showed significantly higher right-side external rotation. Other studies [14-16] show similar values for the ABD of rotation. These IBDs of the rotation should probably be considered during femur/tibial surgery.

However, the IBD of femur and tibia rotation in Japanese patients is unknown because previously presented studies [13-16] with CT analysis showed only the outcomes of American institutes. Interracial differences in the IBD of femur and tibia rotation may exist.

The aim of this study was to evaluate the rotation of the femur, knee, tibia, and leg, sex differences, and the IBD of rotation in Japanese individuals with healthy bones via CT analysis. Knowledge of the IBD of rotation in healthy Japanese individuals may be useful for reducing fractured femurs and tibias during surgery.

## Materials and methods

This retrospective study was conducted at Okayama City Hospital, Okayama, Japan, a single level-two trauma center, in accordance with the Declaration of Helsinki guidelines. Approval was obtained from the Institutional Review Board of Okayama City Hospital prior to the study (approval number: 4-267). Informed consent was obtained in the form of an opt-out on the website.

**Figure 1:**
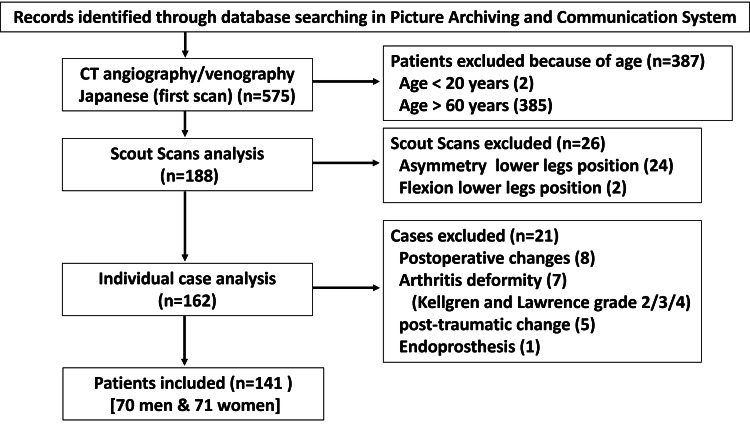
Flowchart of the patient inclusion process CT: computed tomography

All consecutive Japanese patients who underwent lower-limb CT angiography or venography between May 2015 and December 2022 at our institution were identified using the hospital’s Picture Archiving and Communication System (PACS). Patients with complete lower-limb CT angiography or venography images depicting the complete femur and tibia with taluses on both sides were eligible for inclusion. Patients who met at least one of the following criteria were excluded from the study: younger than 20 years or older than 60 years of age; asymmetry or flexion position of the legs on scout scans; osteoarthritis of the hip and knee joint with joint deformity (i.e., Kellgren and Lawrence grade 2/3/4 [17]); endoprosthesis of the hip, knee, or ankle joint; postoperative changes of the lower limb; and post-traumatic changes of the lower limb. A flowchart of the patient inclusion process is shown in Figure 1.

Data setting

Lower-limb CT angiography or venography was used for rotation alignment analysis. The patients were placed in the supine position with extended lower limbs during the standardized CT examination protocol. The CT scans were conducted using a CT system (SOMATOM Force; Siemens, Munich, Germany) with constant reconstruction parameters. Sections of 0.75 mm thickness were reconstructed from the raw data.

Axial orientation was used to measure the radiographic parameters of the femoral and tibial rotations. The measurements were acquired digitally using a picture archiving system (Synapse Radiology Information System; Fujifilm, Tokyo, Japan). Measurements were independently conducted by three orthopedic surgeons (with one year, 10 years, and 20 years of experience, respectively) who were familiar with rotational analyses. The values of the rotation angle were calculated based on the average of the measurements of the three observers.

Definition of measurement axes and measurement of rotation angles

**Figure 2:**
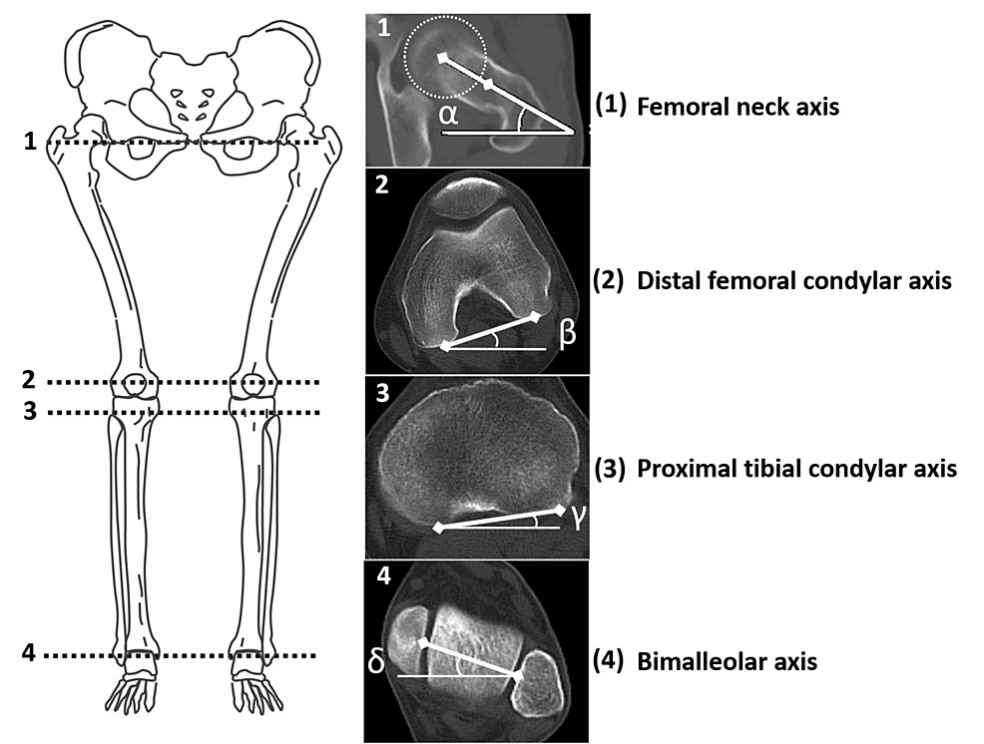
Axial axes of lower limbs and measurements of the rotation angle The angles between the axes at levels 1 to 4 are represented by α, β, γ, and δ, respectively. External rotation angle has a plus value, and internal rotation angle has a minus value. Femur rotation is measured by β - α; knee rotation, γ - β; tibia rotation, δ - γ; leg rotation with knee, δ - α; and leg rotation without knee, (δ-α) - (γ-β). This figure is the authors' original work.

The alignment of femoral and tibial rotation was assessed, following the methodology outlined by Shih et al. [18] and other investigators [19-22]. Four axes (two in the femur and two in the lower leg) were measured. Those were as follows: (1) the femoral neck axis, (2) the distal femoral condylar axis, (3) the proximal tibial condylar axis, and (4) the distal tibial (bimalleolar) axis (Figure 2).

Femoral rotation was measured as the angle formed between the line intersecting the femoral neck and the posterior condylar line (PCL) of the distal femur. The femoral neck axis was defined, according to Reikerås et al. [19], as the line between the center of the femoral head and the neck bisector in two CT cuts where the widest femoral head and the widest neck are evident. Positive values represented femoral retroversion, and negative values represented anteversion of the femoral neck in relation to the PCL.

Tibial rotation was measured as the angle formed between the line connecting the posterior aspects of the proximal tibial condyles and the bimalleolar axis. The line connecting the posterior aspects of the proximal tibial condyle was set at the apex of the fibula [21]. The bimalleolar axis is drawn in a cut just below the articular surface of the tibial pilon, with the medial and lateral malleoli and talar dome evident between the centers of the dense surfaces of the malleoli [22]. The external rotation of the tibia was represented by positive values. Negative values indicated internal rotation of the distal tibia relative to the baseline of the proximal posterior tibial plateau.

Additionally, knee and whole-leg rotation alignment was measured. Knee rotation was measured as the angle formed between the line connecting the posterior aspects of the proximal tibial condyles and the PCL of the distal femur. The external rotation of the knee was represented by positive values. These variables presented elements of knee laxity in the extension position.

Leg rotation with the knee was defined as the overall axial lower limb rotation, based on the angle of the femoral neck axis to the bimalleolar axis. This leg rotation included potential rotational elements, owing to knee laxity.

Leg rotation without the knee was defined as the angle of the overall axial lower limb rotation after removing knee rotation. This leg rotation did not include potential rotational elements, owing to knee laxity.

Statistical analyses

Descriptive statistics were used to describe demographic, rotation, and IBD variables. The variables were tested for normality by using the Shapiro-Wilk test. All variables, except for IBD, were normally distributed. The paired two-sample t-test was used to compare differences in the mean right versus left rotation. The Student's two-sample t-test was used to compare rotation and the IBD of rotation by sex. The significance level for alpha error was set at 0.05. Pearson’s correlation coefficient was calculated for right-left correlation. The IBD of the variables was measured as the right rotation minus the left rotation and was expressed as RBD and ABD. The RBD showed right-left trends, and the ABD showed absolute differences on the right-left sides. For the reliability analysis, intraclass correlation coefficients (ICCs) were obtained by interobserver comparisons of three persons and intraobserver comparisons of two measurements by one observer. Statistical analyses were conducted using EZR analysis software, v1.5 (The R Foundation for Statistical Computing, Vienna, Austria) [23] and Modified R Commander, v4.0.2 (for Windows: https://personal.hs.hirosaki-u.ac.jp/pteiki/research/stat/R/).

## Results

We screened 575 consecutive bilateral lower-limb CT angiography and venography images. A total of 141 patients (70 men and 71 women) included in the study had a mean age of 44.7 years (men: 46.1 years; women: 43.0 years; age range, 20-59 years). The ICCs were between 0.94 and 0.99 for all measurements, indicating excellent reliability (i.e., greater than 0.90) (Table 1) [24].

**Table 1:** Intraclass correlation coefficients Inter (2,1) is the interobserver reliability, based on three observers, and intra (1,2) is the intraobserver reliability, based on two measurements by one observer. CI: confidence interval

	Inter (2,1)	95%CI		Intra (1,2)	95%CI	
Right femoral neck axis	0.95	0.924	0.970	0.99	0.996	0.997
Left femoral neck axis	0.94	0.919	0.956	0.99	0.989	0.994
Right distal femoral condyle axis	0.99	0.994	0.997	0.99	0.999	0.999
Left distal femoral condyle axis	0.99	0.992	0.996	0.99	0.999	0.999
Right proximal tibial condyle axis	0.98	0.986	0.992	0.99	0.999	0.999
Left proximal tibial condyle axis	0.99	0.987	0.992	0.99	0.999	0.999
Right distal tibial (bimalleolar) axis	0.98	0.973	0.988	0.98	0.972	0.985
Left distal tibial (bimalleolar) axis	0.98	0.979	0.988	0.98	0.966	0.983

For all limb analyses (Table 2), the mean femur anteversion (internal rotation) was 17.3°, the mean knee external rotation was 5.5°, the mean tibia external rotation was 25.7°, the mean leg external rotation with the knee was 13.9°, and the mean leg external rotation without the knee was 8.4°. Men had significantly higher external rotation, except for knee rotation. Women with knee rotation had significantly higher external rotation. The frequency of rotation patterns, based on side (Table 3), indicated that femur retroversion (external rotation) was 7.8%, knee internal rotation was 8.9%, tibia internal rotation was 0%, and leg internal rotation with and without the knee was 14.9% and 28.0%, respectively.

**Table 2:** Measurements of all limbs CI: confidence interval; w/o: without ^a^Indicates a statistically significant value. Values are presented as the mean and standard deviation (SD). External rotation is indicated by a plus (+) sign, and internal rotation by a minus (-) sign. The significance level for the alpha error is set at 0.05.

	All (limb: n=282)	Men (limb: n=140)	Women (limb: n=142)	P-value	95%CI	
	(mean (SD) degree)	(mean (SD) degree)	(mean (SD) degree)			
Age (y)	44.7 (9.0)	46.1 (9.7)	43.0 (8.1)	0.044^ a^	-6.0	-0.1
Femur rotation	-17.3 (11.8)	-13.7 (11.6)	-20.8 (11.0)	<0.001^ a^	-9.8	-4.5
Knee rotation	5.5 (4.8)	4.2 (5.0)	6.9 (4.3)	<0.001^ a^	1.6	3.8
Tibia rotation	25.7 (8.7)	27.0 (7.9)	24.6 (9.3)	0.012^ a^	-4.6	-0.6
Leg rotation with knee	13.9 (12.7)	17.4 (12.0)	10.5 (12.5)	<0.001^ a^	-9.8	-4.0
Leg rotation w/o knee	8.4 (13.5)	13.3 (12.8)	3.5 (12.5)	<0.001^ a^	-12.7	-6.7

**Table 3:** Frequency of the rotation patterns based on side

Side	Right (n)	Left (n)
Femur anteversion	131 (93%)	129 (91%)
Femur retroversion	10 (7%)	12 (9%)
Knee internal rotation	14 (10%)	11 (8%)
Knee external rotation	127 (90%)	130 (92%)
Tibia internal rotation	0 (0%)	0 (0%)
Tibia external rotation	141 (100%)	141 (100%)
Leg internal rotation with knee	20 (14%)	22 (16%)
Leg external rotation with knee	121 (86%)	119 (84%)
Leg internal rotation w/o knee	35 (26%)	44 (32%)
Leg external rotation w/o knee	105 (74%)	96 (68%)

The Pearson correlation coefficient of the femur/knee/tibia/leg rotation between the right and left sides showed a high correlation (r = 0.702 to 0.81) for all elements (Table 4). In the all-side analyses (Table 5), the mean ABD of femur rotation was 6.5°, the mean ABD of knee rotation was 2.4°, the mean ABD of tibial rotation was 5.1°, and the mean ABD of leg rotation with and without knees was 8.1° and 8.2°, respectively. The RBD of femoral rotation showed no significant difference between the right and left sides. The RBD of knee rotation showed significantly higher right-sided internal rotation. The RBD of tibia/leg rotation showed significantly higher external rotation on the right side. In the sex-specific side analysis (Table 6), similar outcomes were observed, except for RBD of knee rotation.

**Table 4:** Pearson correlation coefficient of limbs between the right and left sides r: Pearson correlation coefficient; CI: confidence interval The significance level for the alpha error is set at 0.05.

	r	P-value	95%CI	
Femur rotation	0.75	<0.001	0.67	0.82
Knee rotation	0.81	<0.001	0.74	0.86
Tibia rotation	0.778	<0.001	0.70	0.84
Leg rotation with knee	0.702	<0.001	0.61	0.78
Leg rotation w/o knee	0.735	<0.001	0.65	0.80

**Table 5:** Measurements for sides in all patients CI: confidence interval; RBD: relative bilateral difference; ABD: absolute bilateral difference; w/o: without ^a^: Statistically significant difference Values are presented as the mean and standard deviation (SD). External rotation is indicated by a plus (+) sign, and internal rotation by a minus (-) sign. The significance level for the alpha error is set at 0.05.

	Side		Side-to-side		P-value RBD	95%CI RBD	
	Right (n=141)	Left (n=141)	RBD (Right-left)	ABD (Right-left)			
	(Mean (SD) degree)	(Mean (SD) degree)	(Mean (SD) degree)	(Mean (SD) degree)			
Femur rotation	-17.2 (11.8)	-17.4 (11.9)	0.2 (8.4)	6.5 (5.2)	0.763	-1.6	1.2
Knee rotation	5.3 (4.7)	5.8 (4.9)	-0.5 (3.0)	2.4 (1.9)	0.042^ a^	0.1	1.0
Tibia rotation	27.3 (8.9)	24.0 (8.2)	3.3 (5.8)	5.1 (4.2)	<0.001^ a^	-4.2	-2.3
Leg rotation with knee	15.4 (13.0)	12.4 (12.3)	3.0 (9.8)	8.1 (6.3)	<0.001^ a^	-4.6	-1.3
Leg rotation w/o knee	10.1 (13.8)	6.6 (13.0)	3.5 (9.8)	8.2 (6.3)	<0.001^ a^	1.8	5.1

**Table 6:** Measurements of the sides based on sex CI: confidence interval; RBD: relative bilateral difference, ABD: absolute bilateral difference; w/o: without ^a^: Statistically significant difference Values are presented as the mean and standard deviation (SD). External rotation is indicated by a plus (+) sign, and internal rotation by a minus (-) sign. The significance level for the alpha error is set at 0.05.

	Side		Side-to-side		P-value RBD	95%CI RBD	
	Right	Left	RBD (Right-left)	ABD (Right-left)			
	(Mean (SD) degree)	(Mean (SD) degree)	(Mean (SD) degree)	(Mean (SD) degree)			
Femur rotation (men)	-13.9 (11.9)	-13.5 (11.4)	-0.4 (8.6)	6.7 (5.4)	0.72	-2.4	1.7
Femur rotation (women)	-20.4 (10.8)	-21.2 (11.2)	0.8 (8.1)	6.3 (5.1)	0.416	-1.1	2.7
Knee rotation (men)	4.0 (4.9)	4.3 (5.2)	-0.3 (3.2)	2.6 (1.9)	0.414	-1.1	0.5
Knee rotation (women)	6.5 (4.3)	7.2 (4.3)	-0.7 (2.8)	2.2 (1.8)	0.033^ a^	-1.4	-0.1
Tibia rotation (men)	29.0 (8.2)	25.0 (7.1)	4.0 (5.8)	5.5 (4.4)	<0.001^ a^	2.6	5.4
Tibia rotation (women)	25.6 (9.4)	23.1 (9.2)	2.5 (5.7)	4.8 (4.0)	<0.001^ a^	1.2	3.9
Leg rotation with knee (men)	19.0 (12.5)	15.7 (11.5)	3.3 (9.2)	7.7 (5.9)	0.004^ a^	1.1	5.5
Leg rotation with knee (women)	11.8 (12.6)	9.2 (12.2)	2.7 (10.4)	8.5 (6.6)	0.035^ a^	0.2	5.1
Leg rotation w/o knee (men)	15.1 (13.4)	11.4 (12.0)	3.6 (9.1)	7.4 (6.5)	0.002^ a^	1.4	5.8
Leg rotation w/o knee (women)	5.2 (12.6)	1.9 (12.2)	3.3 (10.5)	9.1 (6.0)	0.009^ a^	0.8	5.8

With regard to the baseline descriptive statistics, separated by sex (Table 7), men had a significantly higher external rotation in all elements except knee rotation, and women had a significantly higher external rotation on knee rotation, similar to the all-side analysis findings. The ABD and RBD for all elements were not significantly different between men and women.

**Table 7:** Baseline descriptive statistics are shown separately for male and female patients. CI: confidence interval; RBD: relative bilateral difference (right-left); ABD: absolute bilateral difference (right-left); w/o: without ^a^: Statistically significant difference Values are presented as the mean and standard deviation (SD). External rotation is indicated by a plus (+) sign, and internal rotation by a minus (-) sign. The significance level for the alpha error is set at 0.05.

	Sex		P-value	95%CI	
	Men (n=70)	Women (n=71)			
	(Mean (SD) degree)	(Mean (SD) degree)			
Femur rotation (right)	-13.9 (11.9)	-20.4(10.8)	<0.001^ a^	-10.3	-2.7
Femur rotation (left)	-13.5 (11.4)	-21.2(11.2)	<0.001^ a^	-11.5	-3.9
RBD femur rotation	-0.4 (8.6)	-0.8(8.1)	0.412	-1.6	3.9
ABD femur rotation	6.7 (5.4)	6.3(5.1)	0.719	-2.1	1.4
Knee rotation (right)	4.0 (4.9)	6.5(4.3)	0.001^ a^	1.0	4.1
Knee rotation (left)	4.3 (5.2)	7.2(4.3)	<0.001^ a^	1.3	4.5
RBD knee rotation	-0.3 (3.2)	-0.7(2.8)	0.425	-1.4	0.6
ABD knee rotation	2.6 (1.9)	2.2(1.8)	0.253	-1.0	0.3
Tibia rotation (right)	29.0 (8.2)	25.6(9.4)	0.027^ a^	-6.3	-0.4
Tibia rotation (left)	25.0 (7.1)	23.1(9.2)	0.178	-4.6	0.9
RBD tibia rotation	4.0 (5.8)	2.5(5.7)	0.135	-3.4	0.5
ABD tibia rotation	5.5 (4.4)	4.8(4.0)	0.332	-2.1	0.7
Leg rotation with knee (right)	19.0 (12.5)	11.8(12.6)	<0.001^ a^	-11.4	-3.0
Leg rotation with knee (left)	15.7 (11.5)	9.2(12.2)	0.001^ a^	-10.5	-2.6
RBD leg rotation with knee	3.3 (9.2)	2.7(10.4)	0.707	-3.9	2.6
ABD leg rotation with knee	7.7 (5.9)	8.5(6.6)	0.446	-1.3	2.9
Leg rotation w/o knee (right)	15.1 (13.4)	5.2(12.6)	<0.001^ a^	-14.2	-5.5
Leg rotation w/o knee (left)	11.4 (12.0)	1.9(12.2)	<0.001^ a^	-13.6	-5.5
RBD leg rotation w/o knee	3.6 (9.1)	3.3(10.5)	0.861	-3.6	3.0
ABD leg rotation w/o knee	7.4 (6.5)	9.1(6.0)	0.095	-0.3	3.9

**Figure 3:**
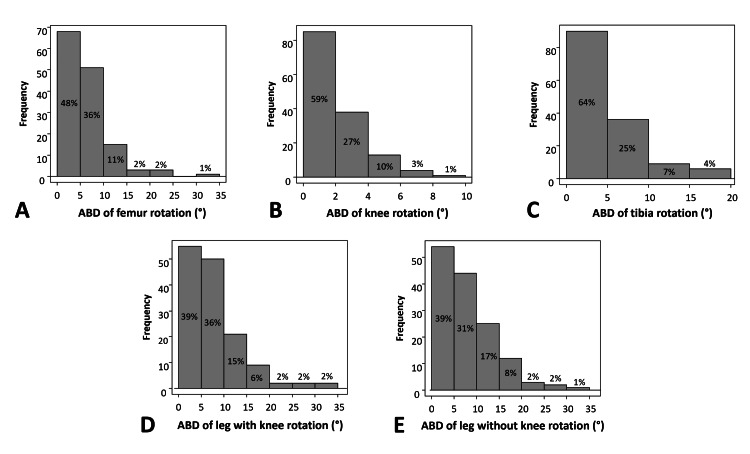
Distribution of the absolute bilateral difference (ABD) of rotation (A) Distribution of ABD of femur rotation; (B) Distribution of ABD of knee rotation; (C) Distribution of ABD of tibia rotation; (D) Distribution of ABD of leg rotation with knee; (E) Distribution of ABD of leg rotation without knee.

With regard to the distribution of ABD (Figure 3), the ABD of femur rotation ≤15° was 95%, the ABD of knee rotation ≤6° was 96%, the ABD of tibia rotation ≤10° was 89%, the ABD of leg rotation with knee ≤20° was 96%, and the ABD of leg rotation without knee ≤20° was 95%.

## Discussion

Summary of results

The aim of this study was to evaluate the rotation of the femur, knee, tibia, and leg, sex differences, and the IBD of rotation among Japanese patients with healthy bones based on CT analysis.

For femur, knee, and tibia rotation, a significant difference was observed between men and women. These differences produced leg rotation differences between men and women. The high correlation between right and left rotation demonstrated that normal side rotation of the femur or tibia can be an indicator of a reduction in the fractured side rotation during surgery. Internal tibial rotation was absent, indicating that internal malrotation was unacceptable during tibial surgery.

The RBD of femoral rotation did not differ significantly between the right and left sides. This trend does not need to be considered during femur surgery. The RBD of the tibia rotation showed a higher mean external rotation of 3.3° on the right side (P<0.001). Side trends should be taken into account during tibial surgery.

The ABD and RBD of rotation for all elements showed no significant differences between male and female individuals. Sex differences need not be considered during surgery, with regard to IBD.

Although leg rotation with and without the knee was analyzed to examine the influence of knee laxity on leg rotation, the outcomes of both methods were similar for all IBD elements in this study.

The distribution of ABD of femur rotation ≤15° was 95%, and ABD of tibia rotation ≤10° was 89%. The outcome supported the previous definition of acceptable rotation differences between the normal and fractured femur and tibia of ≤15° and ≤10°, respectively.

Comparison with previous studies

Recent studies using CT analysis had different outcomes than those of the present study in femur, tibia, and leg rotation, probably because of interracial differences or differences in measurement methods [13-16]. However, previous studies had outcomes similar to those of the present study on IBD caused by rotation. Reis et al. [13] reported outcomes similar to those of the present study on the ABD of femur, tibia, and leg rotation and on the RBD of tibia and leg rotation. Croom et al. [14] reported outcomes similar to those of the present study on the ABD and RBD of femur rotation, and Gallo et al. [15] and Volkmar et al. [16] reported outcomes similar to those of the present study on the ABD and RBD of tibial rotation. Comparisons of previous studies and the present study’s findings on IBDs are shown in Tables 8-10. The aim of this study was to evaluate the rotation of the femur, knee, tibia, and leg, sex differences, and the IBD of rotation among Japanese patients with healthy bones based on CT analysis.

**Table 8:** Comparison of previous studies and the present study’s findings on individual bilateral differences in femur rotation RBD: relative bilateral difference (right-left); ABD: absolute bilateral difference (right-left) ^a^: Statistically significant difference Values are presented as the mean and standard deviation (SD). External rotation is indicated by a plus (+) sign, and internal rotation by a minus (-) sign. The significance level for the alpha error is set at 0.05.

Author	Number of Individuals	Age (y)	Right femur rotation	Left femur rotation	RBD of femur rotation	P-value	ABD of femur rotation
		(Mean)	(Mean (SD) degree)	(Mean (SD) degree)	(Mean degree)		(Mean (SD) degree)
Reis et al. [13]	105	67	-10.1 (9.3)	-12.4 (8.9)	2.2	0.002^ a^	6.0 (4.7)
Croom et al. [14]	164	48.3	-9.7 (9.4)	-9.1 (9.4)	-0.6	0.31	5.4 (4.4)
Present study	141	44.7	-17.2 (11.8)	-17.4 (11.9)	0.2	0.763	6.5 (5.2)

**Table 9:** Comparison of previous studies and the present study’s findings on individual bilateral differences in tibia rotation RBD: relative bilateral difference (right-left); ABD: absolute bilateral difference (right-left) ^a^: Statistically significant difference Values are presented as the mean and standard deviation (SD). External rotation is indicated by a plus (+) sign, and internal rotation by a minus (-) sign. The significance level for the alpha error is set at 0.05.

Author	Number of Individuals	Age (y)	Right tibia rotation	Left tibia rotation	RBD of tibia rotation	P-value	ABD of tibia rotation
		(Mean)	(Mean (SD) degree)	(Mean (SD) degree)	(Mean degree)		(Mean (SD) degree)
Reis et al. [13]	105	67	32.7 (9.9)	29.9 (9.6)	2.3	<0.001^ a^	5.7 (4.8)
Gallo et al. [15]	195	56.5	28.8 (8.9)	26.2 (8.8)	2.6	<0.001^ a^	5.3 (4.0)
Volkmar et al. [16]	229	45.1	37.9	33.4	4.5	<0.001^ a^	6
Present study	141	44.7	27.3 (8.9)	24.0 (8.2)	3.3	<0.001^ a^	5.1 (4.2)

**Table 10:** Comparison of previous studies and the present study’s findings on individual bilateral differences in leg rotation RBD: relative bilateral difference (right-left); ABD: absolute bilateral difference (right-left) ^a^: Statistically significant difference Values are presented as the mean and standard deviation (SD). External rotation is indicated by a plus (+) sign, and internal rotation by a minus (-) sign. The significance level for the alpha error is set at 0.05.

Author	Number of Individuals	Age (y)	Right leg rotation	Left leg rotation	RBD of leg rotation	P-value	ABD of leg rotation
		(Mean)	(Mean (SD) degree)	(Mean (SD) degree)	(mean degree)		(Mean (SD) degree)
Reis et al. [13]	105	67	24.7 (12.0)	19.6 (11.3)	5.1	<0.001^ a^	9.5 (7.6)
Present study	141	44.7	15.4 (13.0)	12.4 (12.3)	3.0	<0.001^ a^	8.1 (6.3)

For femur, knee, and tibia rotation, a significant difference was observed between men and women. These differences produced leg rotation differences between men and women.

The high correlation between right and left rotation demonstrated that normal side rotation of the femur or tibia can be an indicator of a reduction in the fractured side rotation during surgery. Internal tibial rotation was absent, indicating that internal malrotation was unacceptable during tibial surgery.

The RBD of femoral rotation did not differ significantly between the right and left sides. This trend does not need to be considered during femur surgery. The RBD of the tibia rotation showed a higher mean external rotation of 3.3° on the right side (P<0.001). Side trends should be taken into account during tibial surgery.

The ABD and RBD of rotation for all elements showed no significant differences between male and female individuals. Sex differences need not be considered during surgery with regard to IBD.

Although leg rotation with and without the knee was analyzed to examine the influence of knee laxity on leg rotation, the outcomes of both methods were similar for all IBD elements in this study.

The distribution of ABD of femur rotation ≤15° was 95%, and ABD of tibia rotation ≤10° was 89%. The outcome supported the previous definition of acceptable rotation differences between the normal and fractured femur and tibia of ≤15° and ≤10°, respectively.

However, Reis et al. [13] reported a different outcome from that of the present study on the RBD of femoral rotation in that they showed a significantly higher right external rotation (Table 8). The mean age of the study population was 67 years, and the exclusion criteria included Kellgren and Lawrence grade 4 osteoarthritis only. Furthermore, the study did not refer to leg position in terms of symmetry. Compared with the measurement of tibial rotation, the measurement of femur rotation is more easily affected by position (i.e., flexion, extension, adduction, or abduction) because of the neck shaft angle [25]. Symmetry and extension positions of the legs are necessary, especially for the measurement of IBD during femur rotation. The Reis study’s measurements may have been affected by the asymmetrical position due to osteoarthritis. On the other hand, Croom et al. [14] report that the mean age in their study was 44 years, which was the same outcome as that of the present study, in which the RBD of femoral rotation had no significant difference between the right and left sides.

Strengths

The interpretation of rotation analysis requires standardized and reliable methods. Reliable landmarks were identified, following a strict protocol based on validated methods. With regard to the identification of reliable landmarks by the three surgeons, our ICCs of interobserver and intraobserver showed high reliability.

We prospectively measured leg rotation using three-dimensional CT (3DCT) data showing true rotations. The measurement of leg rotation using two-dimensional CT (2DCT) is affected by position (i.e., flexion, extension, adduction, and abduction). The 3DCT data could not be obtained retrospectively from the PACS. Measurements based on symmetry and extension position of the leg by 2DCT probably show similar values to those of 3DCT, at least with respect to IBD.

Limitations

First, selection bias may have occurred in patients undergoing CT angiography or venography. They do not represent the general healthy population because they may have diseases that require angiography or venography.

Second, the judgment of the patients’ Japanese origin, based only on the patients’ names, may have caused a measurement bias. Individuals with other origins who became naturalized as Japanese may have been included.

Third, interracial differences between the present and previous studies could not be directly compared. Therefore, measuring and comparing data using the same measurement methods for populations of different races is necessary.

## Conclusions

The distributions of ABD in femur and tibia rotation supported the previous definition of an acceptable rotation difference between normal and fractured femur and tibia of ≤15° and ≤10°, respectively. During tibial surgery, surgeons should take into account that, compared with the left side, the right side may have a higher degree of external rotation. The IBD of femur and tibia rotation in the Japanese population was similar to that in American studies. The IBD of rotation was not significantly different between male and female individuals. Interracial and sex-based differences need not be considered with regard to IBD. To connect these findings to clinical benefits, we plan to develop reliable methods for measuring the rotation of the femur and tibia during surgery.
